# NF1 expression profiling in IDH-wildtype glioblastoma: genomic associations and survival outcomes

**DOI:** 10.1186/s40478-024-01875-z

**Published:** 2024-10-29

**Authors:** Michael Chang, Mohamed Sherief, Maria Ioannou, Viveka Chinnasamy, Lucy Chen, Michael Frost, Michelle Mattson-Hoss, Herb Sarnoff, David O. Kamson, Matthias Holdhoff, Debraj Mukherjee, Chetan Bettegowda, Jordina Rincon-Torroella, Victoria Croog, Peng Huang, Fausto J. Rodriguez, Calixto-Hope G. Lucas, Karisa C. Schreck

**Affiliations:** 1 Department of Neurology, Johns Hopkins University School of Medicine, Baltimore, MD USA; 2 Infixion Bioscience, Inc., San Diego, CA USA; 3 Department of Oncology, Johns Hopkins University School of Medicine, Baltimore, MD USA; 4 Department of Neurosurgery, Johns Hopkins University School of Medicine, Baltimore, MD USA; 5 Department of Radiation Oncology, Johns Hopkins University School of Medicine, Baltimore, MD USA; 6 Department of Biostatistics, Johns Hopkins Bloomberg School of Public Health, Baltimore, MD USA; 7 Division of Neuropathology, University of California, Los Angeles, Los Angeles, CA USA; 8 Department of Pathology, Johns Hopkins University School of Medicine, Baltimore, MD USA

**Keywords:** Neurofibromin, NF1, Biomarker, Glioblastoma, High-grade glioma

## Abstract

**Background:**

NF1 inactivation is associated with sensitivity to MEK inhibitor targeted therapy in low-grade and some high-grade gliomas. NF1 loss may also be a harbinger of exploitable vulnerabilities in IDH-wildtype glioblastoma (GBM). Accurate and consistent detection of NF1 loss, however, is fraught given the large gene size, challenges with complete coverage and variant calling upon sequencing, and mechanisms of mRNA and protein regulation that result in early degradation in the absence of genomic alterations. Here, we seek to perform a composite analysis for NF1 loss accounting for genomic alterations and protein expression via immunohistochemistry. We also characterize the landscape of *NF1* alterations in GBM.

**Methods:**

We assembled a single-institution, retrospective cohort of 542 IDH-wildtype GBM with somatic next generation sequencing to investigate the frequency and nature of detected *NF1* alterations. We selected 69 GBMs from which to build a tissue microarray (TMA) of 44 *NF1*-wildtype and 25 *NF1*-mutant cases. We performed NF1 immunohistochemistry using two different NF1 antibodies (NFC, Sigma-Aldrich; and iNF-07E, iNFixion Bioscience) and correlated results with clinical, genomic, and other immunohistochemical features.

**Results:**

In our retrospective cohort, we identified 88 IDH-wildtype GBM with *NF1* alterations (16%). *NF1* alterations were mutually exclusive with *EGFR* and *MDM2* alterations (*p*-adj < 0.001, 0.05, respectively), but co-occurred with *PIK3R1* alterations (Log_2_(OR) = − 1.6, p-adj = 0.03). Of the 63 scorable sporadic GBMs in the TMA, 14 harbored *NF1* inactivating alterations and of those, 12 (86%) demonstrated minimal NF1 immunoreactivity by NFC antibody, compared to 8 (57%) by iNF-07E antibody. Among the 42 scorable *NF1*-wildtype GBM in the TMA, NF1 immunostaining was minimal in 18 (43%) by NFC antibody compared to 4 (10%) by iNF-07E antibody, potentially reflecting false positives or differential protein regulation. Minimal immunoreactivity by NFC antibody was associated with decreased median overall survival (8.5 vs. 16.4 months, *p* = 0.011). Cox proportional hazards model correcting for prognostic variables in this subset revealed HR 3.23 (95% CI 1.29–8.06, *p* = 0.01) associated with decreased NF1 expression by IHC.

**Conclusion:**

NF1 immunostaining may serve as a sensitive surrogate marker of *NF1* genomic inactivation and a valuable extension to next-generation sequencing for defining NF1 status. Minimal NF1 immunoreactivity is a poor prognostic marker, even in IDH-wildtype glioblastoma without apparent *NF1* genomic alterations, but the underlying molecular mechanism requires further investigation.

**Supplementary Information:**

The online version contains supplementary material available at 10.1186/s40478-024-01875-z.

## Introduction

IDH-wildtype glioblastoma (GBM) is the most common primary brain malignancy in adults [1]. The prognosis is poor, and the treatment paradigm—a combination of surgical resection, radiotherapy and alkylating chemotherapy—has remained largely unchanged for the past two decades [2]. Advances in tumor genomics have identified clinically relevant genetic alterations and epigenetic signatures in GBM, but O (6)-methylguanine-DNA methyltransferase (*MGMT*) promoter methylation remains one of the few molecular markers predictive of response to standard therapy [3]. Some oncogenic mutations have proven to be actionable clinical targets in other glioma types. Successful trials of IDH-inhibitors for IDH-mutant astrocytoma, BRAF inhibitors for BRAF-mutant glioma, and MEK inhibitors for pediatric low-grade glioma have demonstrated the potential impact of targeted therapy for gliomas, but unfortunately not yet in GBM [4–6]. Identification of targetable oncogenes and development of effective targeted therapies for GBM remains an area of active investigation.

Neurofibromin 1 *(NF1)* is a potentially targetable gene in the MAPK pathway. NF1 is a tumor suppressor that functions as a RAS GTPase activating protein, negatively regulating RAS/MAPK signaling by promoting the inactive GDP-bound form of RAS. Germline mutations in *NF1* cause neurofibromatosis type 1 (NF-1), a tumor predisposition syndrome associated with benign and malignant tumors primarily in the peripheral and central nervous system. Somatic mutations in *NF1* are implicated in many sporadic cancers, including melanoma, lung cancer, ovarian cancer, and GBM [7]. In GBM, *NF1* loss is more common at recurrence and is associated with the mesenchymal subgroup, which has the worst clinical outcomes [8]. NF1 inactivation has been shown to occur through multiple mechanisms. Fifteen percent of sporadic GBM harbor somatic mutations in *NF1* [8]. In addition to truncating and inactivating mutations, NF1 inactivation occurs through other mechanisms such as aberrant splicing, post-transcriptional silencing, and proteasomal degradation [9–12]. Preclinical studies from several groups, including our own, demonstrate that NF1 inactivation confers ERK pathway dependence and partial sensitivity to MEK inhibition [13, 14]. Early-phase clinical trials in pediatric low-grade glioma and case studies in high-grade glioma confirm putative sensitivity to MEK inhibitor therapy [15, 16]. However, effective clinical translation of promising targeted therapy combinations is stymied by challenges in accurately identifying tumors with functional NF1 loss.

Accurately and consistently identifying loss of NF1 function through next-generation sequencing is challenging for several reasons. The gene is large (350 kbp) and can be difficult to sequence in its entirety at sufficient depth for variant calling [17, 18]. Additionally, the functional implications of single nucleotide variants (SNVs) in *NF1* are largely unclear, and non-coding intronic or promoter alterations may not be detected via standard clinical exome sequencing. Moreover, next generation sequencing (NGS) does not account for post-transcriptional modifications, silencing, or degradation which may lead to functional loss of NF1. Here, we evaluate the genomic landscape of *NF1* alterations in IDH-wildtype glioblastoma, validate an immunohistochemical biomarker for NF1 loss, and identify other immunohistochemical, genomic, and clinical correlates of NF1 immunostaining.

## Methods

### Database construction

This study was conducted with the approval of the Johns Hopkins Institutional Review Board (IRB00288357). A REDCap database was constructed from 542 patients with histologically-confirmed IDH-wild type GBM, including 8 gliosarcomas, seen at the Johns Hopkins medical system between November 1, 2017, and December 1, 2023. All tumor specimens underwent sequencing of ~ 400 cancer-related genes via the JHH (Johns Hopkins Hospital) NGS Solid tumor panel, as previously described [19, 20]. For each patient, clinical and next-generation sequencing data were extracted from the electronic medical record. *NF1* genomic inactivation status was determined by a neuropathologist based on the *NF1* alteration(s) and comparative variant allele frequencies.

### Tissue microarray construction

A tissue microarray was constructed using formalin-fixed, paraffin-embedded (FFPE) tissue from a subset of patients comprising the REDCap database. Four, 0.6 mm tumor cores were selected from each specimen based on the microscopic appearance of an adjacent tissue slide as evaluated by a neuropathologist. Regions of high tumor density and cell viability were prioritized for core selection. The final tissue microarray included 69 tumors – 44 *NF1*-wildtype and 25 *NF1*-mutant—including one from a patient with NF-1. Tumors were selected from patients who had an initial diagnosis of glioblastoma, IDH-wildtype after November 1, 2017, and had ample surgical tissue available per visual review of paraffin blocks. Following processing and staining, all specimens except one had two or more evaluable cores.

### Analysis of publicly available glioblastoma cohorts

The cBioportal website (cbioportal.org) was used to analyze The Cancer Genome Atlas (TCGA)—Firehose Legacy and the National Cancer Institute’s Clinical Proteomic Tumor Analysis Consortium (CPTAC) Glioblastoma cohorts [21–24]. We excluded 7 and 15 samples, respectively, from the TCGA and CPTAC cohorts that harbored pathogenic *IDH1/2* alterations detected on NGS.

### Immunohistochemistry

We validated two anti-NF1 antibodies—NFC (Sigma-Aldrich, MABE1820) and iNF-07E (iNFixion Bioscience) in FFPE cell pellet sections made from glioblastoma neurosphere lines with previously characterized levels of NF1 expression: JHH-520 (NF1-deficient), GBM1 (NF1-intact), and JHH-0879 (NF1-intact) [13] (Supplementary Fig. 1). The NFC monoclonal antibody recognizes a C-terminal epitope within the last 281 amino acids of human neurofibromin [25]. iNF-07E is a monoclonal antibody that recognizes amino acids 863–867 of NF1. Both antibodies were validated first in human glioma cells with known NF1 expression status. Immunohistochemical (IHC) staining was performed in FFPE tissue microarray sections using the following antibodies against NF1: clone NFC (1:10) and iNF-07E (1:20), podoplanin (clone D2-40, Dako, 1:200), phospho-ERK (#9101, Cell Signaling, 1:250), phospho-S6, p16, ATRX, Ki67, and p53. At least two scorable tissue cores per specimen were required for score assignment. Scoring for NF1, podoplanin, phospho-ERK, phospho-S6, and p16 was performed by two blinded, independent neuropathologists (F.J.R., C.H.L.) using a three-tiered scale (“preserved”, “equivocal”, “lost”) based on staining intensity. Tumors with discordant scores were assigned an aggregate score of “lost” if at least one core was “lost.” Tissue microarray sections were stained for p53 and Ki67 and digitally scanned. QuPath (v0.4.3) was used to quantify the percentage of cells with positive or negative immunostaining [26].

### Statistical analyses

Association between two categorical variables was summarized using contingency tables and analyzed using two-sided Fisher’s Exact test. To account for multiple comparisons, p-values are adjusted using Benjamini–Hochberg procedure to control false discovery rate of 0.05. Overall survival time was defined from the date of initial surgical diagnosis to the date of death (non-censored) or the date of last known alive (censored). Survival rate was estimated using Kaplan–Meier estimator and compared using log-rank test in univariate analysis. Cox proportional hazards model was used in multivariate analysis. Statistical significance was set at *p* < 0.05. All statistical analyses were performed in R (version 4.3.1) using the ‘survival’, ‘survminer’, and ‘stats’ packages.

## Results

### Characterization of genomic NF1 status in a glioblastoma cohort

**Fig. 1:**
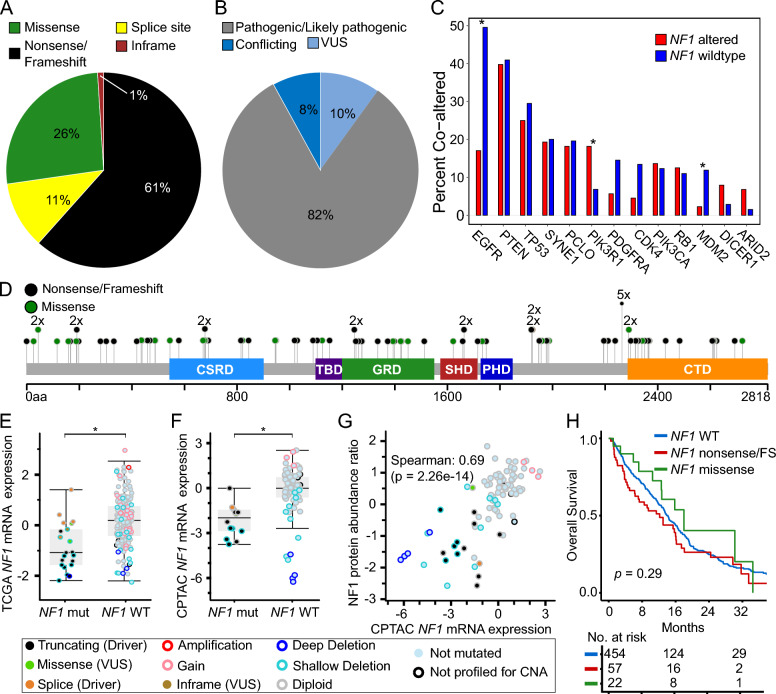
Genomic and clinical features of NF1 loss in glioblastoma. **A** Distribution of *NF1* alteration types and **B** ClinVar classification of *NF1* alterations detected in JHH GBM cohort (N = 115). **C** Co-alteration frequencies between *NF1* and other selected genes in JHH GBM cohort. Statistical significance was assessed using two-tailed Fisher’s Exact test. **D** Lollipop plot of *NF1* alterations detected in JHH GBM cohort. Box and whisker plots comparing *NF1* mRNA expression between *NF1* mutant and *NF1* wildtype glioblastomas in **E** TCGA (z-scores, U133 microarray) and **F** CPTAC (z-scores, log_2_FPKM) GBM cohorts. Statistical significance was assessed using Welch’s *t*-test. **G** Scatter plot comparing NF1 protein abundance ratio (z-scores) and *NF1* mRNA expression (z-scores, log_2_FPKM) in CPTAC GBM cohort. **H** Kaplan–Meier survival curves displaying overall survival in JHH GBM cohort stratified by *NF1* alteration type. VUS = variant of unknown significance, FS = frame shift

We first assessed the frequency and type of *NF1* alteration among 542 IDH-wildtype GBM. One hundred fifteen *NF1* alterations were identified across eighty-eight tumors (16.2%) (Supplementary Table 1). Seventy-one nonsense/frameshift mutations, 13 splice-site mutations, 30 missense mutations, and one in-frame insertion/deletion in *NF1* were annotated (Fig. 1A). Protein-coding changes in *NF1* were distributed throughout the entire gene, with the exception of recurrent truncations at Y2264 which were observed in five tumors. Seventy-two *NF1* alterations were identified by ClinVar to be either “Pathogenic” or “Likely pathogenic”, with 16 alterations of conflicting or uncertain significance and 27 not annotated [27]. Of the 30 *NF1* missense mutations detected in our cohort, seven were predicted to be cancer-promoting and the rest were predicted to be likely passenger mutations by FATHMM [28].

*NF1* alterations were mutually exclusive with alterations in *EGFR* (log2OR –2.3, p-adj < 0.001)*, MDM2* (log2OR –2.5, p-adj = 0.05)*, PDGFRA,* and *CDK4* (Fig. 1B, Supplementary Table 2). Alterations in *NF1* co-occurred with alterations in *PIK3R1* (log2OR 1.6, p-adj = 0.03). After adjusting for multiple hypotheses, only the associations between *NF1* and *EGFR*, *PIK3R1*, and *MDM2* remained statistically significant, supporting prior observations [8]. Mutual exclusivity of *NF1* and *EGFR* alterations in IDH-wildtype glioblastoma was further substantiated using two publicly available resources, TCGA and CPTAC, totaling 368 genomically-characterized glioblastomas after excluding samples with pathogenic *IDH1/2* alterations (Supplementary Table 3–4) [21–24].

To identify whether pathogenic alterations in *NF1* are associated with changes in mRNA or protein expression, we interrogated the TCGA and CPTAC glioblastoma cohorts [21, 22]. We observed significantly decreased average *NF1* mRNA expression (*p* < 0.001 for each cohort) in tumors with known pathogenic *NF1* alterations, though a subset had intact expression levels (Fig. 1E, F). We next evaluated whether *NF1* mRNA expression correlated with protein expression. In 89 glioblastoma samples with both protein and mRNA, mRNA expression levels strongly correlated with protein expression (Spearman = 0.69, *p* < 0.001) as quantified by mass spectrometry (Fig. 1G).

We evaluated how *NF1* alterations were associated with overall survival. In our institutional cohort of 542 glioblastomas, we observed that neither truncating *NF1* alterations in the entire cohort nor *NF1* genomic loss in the TMA cohort alone were significantly associated with decreased overall survival (Fig. 1H). This was supported by the TCGA dataset where neither *NF1* alterations nor mRNA expression was associated with a difference in overall survival (Supplementary Fig. 2A-B). Our findings contrasted with the CPTAC dataset where *NF1* alterations were associated with decreased overall survival, though this was not true for *NF1* mRNA or protein expression (Supplementary Fig. 2C-E) [23, 24].

### Tissue microarray assembly and genomic characterization

**Fig. 2:**
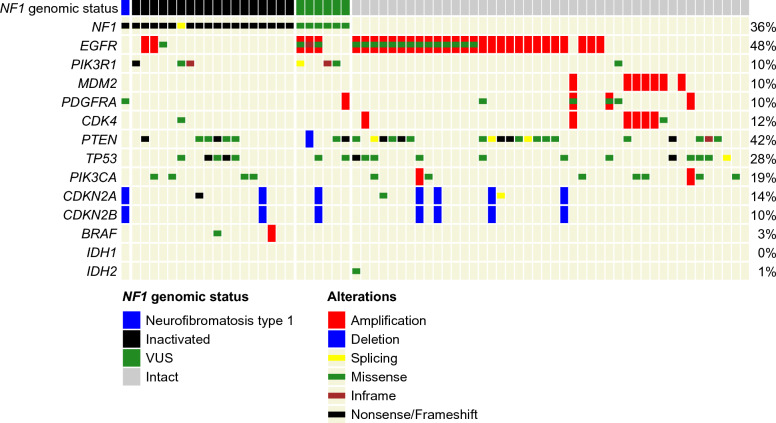
Oncoprint of 69 glioblastomas included in TMA cohort displaying genetic alterations in selected genes

In order to evaluate NF1 protein expression, we created a tissue microarray (TMA) from 69 representative glioblastomas from the cohort described above. Forty-four samples were *NF1*-wildtype and 25 were *NF1*-mutant on next-generation sequencing. All but one was obtained from the first tumor resection. The mutational profiles of glioblastomas included in the tissue microarray cohort are displayed in Fig. 2. The range of genomic alterations and their association with NF1 loss were similar to the overall GBM population. *NF1* alterations identified in our cohort were manually assigned to two categories (pathogenic or non-pathogenic) by a neuropathologist based on whether they were indicative of genomic loss, taking into account alteration type, location in the gene, and potential loss of heterozygosity events. Tumors in the TMA were categorized as “*NF1* altered” or “*NF1* intact” by a neuropathologist based on their alterations. These scores were in high concordance (100%) with ClinVar annotations.

### Immunohistochemistry for NF1 status

**Fig. 3:**
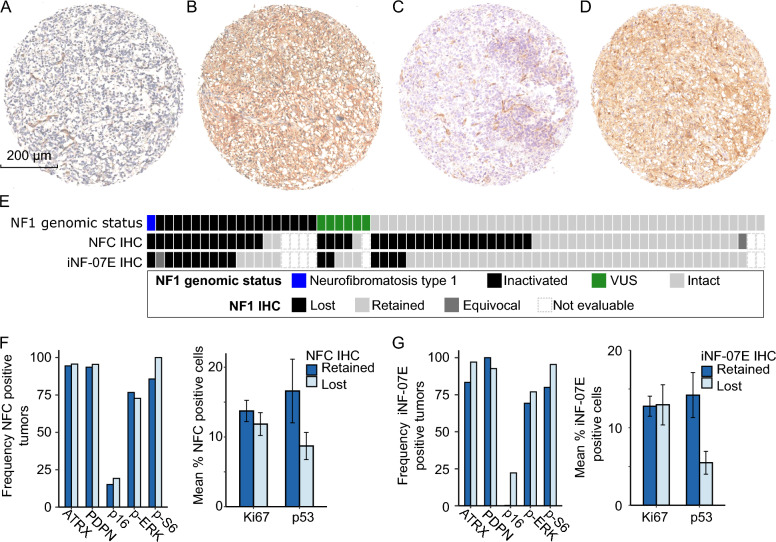
NF1 immunohistochemistry in patient-derived glioblastoma samples. Representative IHC shows loss of NF1 immunostaining in *NF1* −/− tumor (patient with neurofibromatosis type 1) with **A** NFC antibody and **C** iNF-07E antibody. Representative IHC shows retained NF1 immunostaining in *NF1* +/+ tumor with **B** NFC and **D** iNF-07E. **E** Plot of *NF1* genomic status and corresponding NF1 IHC scores using two NF1 antibodies. **F**, **G** I Bar plots demonstrating no difference in expression of candidate surrogate biomarkers selected a priori—phospho-ERK, phospho-S6, p16, podoplanin (PDPN), ATRX, Ki-67, p53—between tumors with retained and lost NF1 immunostaining

We performed an exhaustive literature search for NF1 antibodies previously validated for immunochemistry in human glioma tissue. After thorough validation, we chose two antibodies: clone NFC (Sigma Aldrich, MABE1820) and iNF–07E (Infixion Biosciences) to evaluate NF1 protein expression (Fig. 3, Supplementary Fig. 3). Another NF1 antibody, clone McNFn27b (Abnova, NB300-154), was tested but not used due to high background staining in our hands. Immunostaining with the NFC antibody was negative in the tissue specimen from a patient with NF-1 (Fig. 3A), but immunoreactive in a specimen with no *NF1* alterations (Fig. 3B). The NFC immunostain was immunoreactive in 26 tumors (41%), whereas 34 (54%) had minimal or absent staining, one had equivocal staining, and seven were not scored due to the absence of assessable tumor tissue. Inter-core heterogeneity was noted within tumors, with seven displaying a mixture of cores showing both retained and absent NFC immunoreactivity. NF1 immunostaining scored by a second independent neuropathologist (F.J.R.) was highly concordant (82%). NF1 immunostaining results were evaluated for each tumor in the context of *NF1* genomic status (Fig. 3E). Of the 14 scorable tumors assessed to be *NF1*-deficient by NGS, 12 (86%) also demonstrated minimal to absent NF1 immunostaining with NFC antibody. Of the two tumors with intact NF1 immunostaining, one harbored an E2624* truncating mutation near the C terminus. This truncation preserves a portion of the NFC antibody epitope and, of note, was the most C-terminal truncation present in our cohort. Among scorable *NF1-*wildtype specimens, the NFC antibody was immunoreactive in 23/42 (55%), equivocal in one (2%), and minimal or absent in 18 (43%). In tumors harboring *NF1* variants of uncertain significance, NF1 immunostaining was minimal or absent in four and immunoreactive in one.

The iNF-07E antibody was appropriately negative in the tissue specimen from a patient with NF-1 (Fig. 3C), but immunoreactive in a specimen with no NF1 alterations (Fig. 3D). The iNF-07E antibody was immunoreactive in 46 tumors (76%). Fourteen (23%) demonstrated minimal or absent immunostaining, one stained equivocally, and seven were not scored due to the absence of assessable tumor tissue. Inter-core heterogeneity was minimal with iNF-07E immunostaining, as no tumors exhibited cores with both intact and absent staining. Of the 14 scorable tumors considered *NF1*-deficient by NGS, eight (57%) also demonstrated minimal or absent immunostaining with iNF-07E. Among scorable *NF1* wildtype specimens, NF1 immunostaining was retained in 38 (90%) and minimal or absent in 4 (10%). In tumors harboring *NF1* variants of uncertain significance, NF1 immunostaining was minimal or absent in two and retained in three.

Upon comparison, the sensitivity of NFC and iNF-07E for tumors with confirmed NF1 loss was 86% and 57%, respectively (Supplementary Table 5).

### Protein and genomic correlates of NF1 loss

We next evaluated potential protein correlates for NF1 loss as identified via immunostaining or NGS. We assessed the expression of proteins known to be correlated with MAPK signaling (phospho-ERK, phospho-S6) or with *NF1* status (p16, podoplanin) [29, 30]. We also evaluated proteins associated with proliferation and loss of cell cycle regulation in glioblastoma (p53, Ki-67) [30]. Expression of these proteins did not correlate with NFC or iNF-07E staining. We next asked whether IHC would be associated with *NF1* genomic status and found positive phospho-ERK immunostaining to be associated with *NF1* genomic loss (*p* = 0.045, Supplementary Table 5), supporting prior observations of a similar correlation in mesenchymal GBM [21]. Unsupervised hierarchical clustering by these seven proteins of interest did not identify distinct clusters corresponding with NF1 immunostaining (Supplementary Fig. 4). We evaluated for co-occurring or mutually exclusive gene alterations and found no statistically significant associations with NF1-deficiency by IHC staining.

### Survival analysis of tissue microarray cohort stratified by NF1 immunostaining

**Fig. 4:**
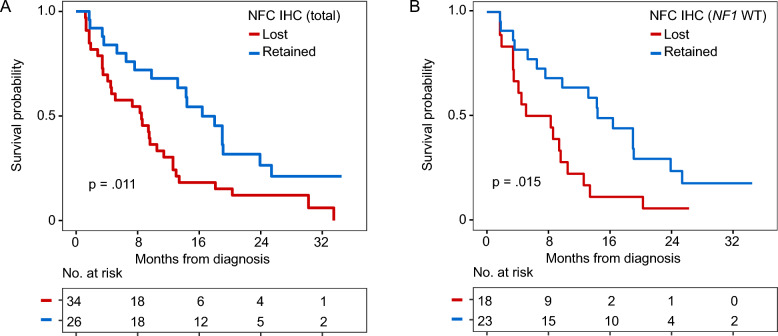
Kaplan–Meier curves displaying overall survival in TMA cohort by NF1 immunohistochemistry. Curves are stratified by **A** NFC immunostaining, **B** iNF-07E immunostaining among entire TMA cohort

We evaluated whether NF1 loss by IHC—as assessed by two different antibodies—correlated with survival. Baseline demographic features of tumors included in the TMA were comparable between the two groups, demonstrating no differences between the two antibodies except for higher baseline Karnofsky performance score observed in patients with loss of immunostaining by iNF-07E antibody (*p* = 0.025) (Supplemental Table 7). Notably, patients with NF1 loss by NFC immunostaining had decreased survival compared with NF1 intact tumors (8.5 (4.5–12.6) vs. 16.4 (13.2–25.4) months, *p* = 0.011; Fig. 4A). This difference was not observed with the iNF-07E antibody (9.8 (6.5—18.0) vs. 11.4 (4.5—NR) months, *p* = 0.63). In a Cox proportional hazard regression model, we adjusted for known and potential confounding variables: age at diagnosis, sex, *MGMT* promoter methylation, baseline Karnofsky performance score, treatment with temozolomide or radiotherapy, extent of resection, and the presence of any *NF1* genomic alteration (Supplementary Table 8). Minimal or absent NFC immunostaining remained significantly associated with decreased overall survival (HR 3.23, 95% CI 1.29–8.06, *p* = 0.01).

### Protein and genomic correlates NF1 expression in *NF1*-wildtype subset

We evaluated whether loss of NF1 expression in *NF1*-wildtype tumors was associated with other gene alterations on NGS or IHC stains. We focused our analysis on the subset of *NF1-*wildtype tumors that demonstrated minimal to absent staining by NFC antibody. None of the genes or proteins that we tested were significantly associated with NF1 expression in this subset (Supplementary Tables 9–10). Notably, despite the absence of associated gene alterations, minimal or absent NF1 protein by NFC remained significantly associated with decreased overall survival in patients without genomic *NF1* alteration (Fig. 4B). Loss of NF1 expression remained significantly associated with decreased survival on multivariate analysis including age at diagnosis, KPS, degree of resection, prior chemoradiation, and *MGMT* promoter methylation status (HR 2.55, 95% CI 1.00–6.49, *p* = 0.049, Supplementary Table 11). On univariate analysis, age at diagnosis, KPS less than 80, subtotal resection, lack of radiation/temozolomide, and unmethylated *MGMT* promoter were also associated with inferior survival, but no other immunostain or genomic alteration was significantly associated with outcome.

## Discussion

NF1 loss is a marker of RAS/MAPK signaling dysregulation and a putatively targetable event in gliomas, but rapid, accurate identification remains fraught. Here, we describe the landscape of *NF1* alterations in a large institutional cohort of 542 molecularly profiled IDH-wildtype glioblastomas, identifying clinical and molecular correlates of NF1 loss. We then evaluated two immunohistochemical antibodies in glioblastoma tissue. Both were effective at identifying NF1 protein loss in tumor specimens predicted to have genomic loss of NF1 but may also identify a subset of *NF1*-wildtype tumors with protein loss.

The mesenchymal subtype of glioblastoma, defined by frequent *NF1* alterations and low mRNA expression of *NF1*, is associated with a poor prognosis [8, 31, 32]. Cells within the mesenchymal subtype are also characterized by hypoxia and an abundance of immune cell infiltrates, and may be present as a subset of cells in a heterogenous glioblastoma [33]. Analysis of a separate institutional cohort of IDH-wildtype glioblastomas also identified *NF1* alterations to be associated with decreased overall survival [34]. Here, we evaluated the public literature supporting the role of *NF1* as a prognostic biomarker and found that the presence of an alteration does not correlate with survival in IDH-wildtype glioblastoma. Even when restricted to known pathogenic truncating alterations, no clear survival difference emerged. While mRNA does correlate with survival, it is costly and time-consuming. In this study, loss of NF1 immunostaining by NFC antibody correlated negatively with survival, regardless of genomic status.

NF1 status is challenging to evaluate via standard next-generation sequencing for many reasons. The *NF1* gene is both large (350 kbp) and structurally complex (60 exons), making it difficult to sequence accurately in its entirety [35]. Targeted next-generation sequencing panels may also fail to report *NF1* structural and copy number variants as well as alterations in noncoding regions, such as promoter or intronic alterations which are known to impact expression of the functional protein [7, 36]. Furthermore, *NF1* has few recurrent or hotspot mutations [7]. Instead, like other tumor suppressor genes, NF1 possesses a diverse mutational spectrum with over 13,000 unique somatic mutations reported in the Catalogue of Somatic Mutations in Cancer [37, 38]. For many of these somatic mutations—especially missense and other non-truncating mutations—their functional consequence is poorly understood [7]. Here we evaluated the utility of an immunohistochemistry-based approach for identifying NF1 loss. The NFC antibody demonstrated minimal immunoreactivity in 86% of tumors with known *NF1* genomic inactivation. It was immunoreactive in one tumor with an *NF1* truncating mutation that was distal to the antibody recognition site (Supp Fig. 3). These findings highlight the importance of interpreting the IHC in concordance with the genomic picture.

Interestingly, both antibodies identified a subset of glioblastoma that was apparently genomically intact for *NF1* but demonstrated minimal to absent protein expression—the NFC antibody more so than iNF-07E (18/42 vs. 4/42). While the current study did not assess *NF1* mRNA for correlation, this may indicate a subset of glioblastoma is inactivating NF1 post-transcriptionally through mechanisms such as proteasomal degradation or post-transcriptional silencing, or through other negative feedback mechanisms. Prior studies suggest that as many as 30% of neurofibromatosis type 1-associated missense mutations in *NF1* result in aberrant pre-mRNA splicing which may explain the observed loss of NF1 protein in these specimens [39]. Validation studies with a larger cohort of glioblastomas having known mRNA expression levels and genomic status will be necessary to further evaluate this observation and determine the underlying mechanism driving minimal NF1 expression.

Overall, these findings highlight the potential value of NF1 immunostaining as a sensitive surrogate marker of NF1 genomic inactivation. We also show that minimal to absent NF1 immunoreactivity is a poor prognostic marker in IDH-wildtype glioblastoma, but the underlying molecular mechanisms leading to decreased NF1 expression in *NF1*-wildtype GBM require further investigation.

## Supplementary Information

Supplementary Figure 1. IHC validation of NF1 antibodies. **(A)** Absent NF1 immunostaining with NFC antibody in JHH-520 (*NF1* -/-) neurosphere cells mixed with NF1-intact B76 filler cell line. **(B,C)** Retained NF1 immunostaining with NFC in GBM1 and JHH-0879 neurosphere cell pellets, both with intact NF1. **(D)** Absent NF1 immunostaining with iNF-07E antibody in JHH-520 neurosphere cells mixed with NF1-intact B76 filler cell line. **(E,F)** Retained NF1 immunostaining with iNF-07E antibody in GBM1 and JHH-0879 neurosphere cell pellets admixed with NF1-intact B76 filler cell line. **(G)** Absent and **(H)** retained NF1 immunostaining with iNF-07E antibody in isogenic NF1 -/- and NF1 +/+ immortalized human Schwann cells respectively.

Supplementary Figure 2. Overall survival and *NF1* alterations, mRNA, and protein expression in TCGA and CPTAC GBM cohorts. Kaplan-Meier curves displaying overall survival in TCGA cohort stratified by **(A)** presence of *NF1* alteration and **(B)** *NF1* mRNA expression (U133 microarray)– bottom quartile versus top quartile. Kaplan-Meier curves displaying overall survival in CPTAC cohort stratified by **(C)** presence of *NF1* alteration, **(D)** *NF1* mRNA expression – bottom quartile versus top quartile, and **(E)** NF1 protein expression – bottom quartile versus top quartile.

Supplementary Figure 3. Lollipop plot of *NF1* alterations identified in TMA cohort with labeled NF1 immunohistochemistry results. iNF-07E antibody epitope and NFC antibody immunogen are labeled in brackets (red).

Supplementary Figure 4. Unsupervised hierarchical clustering of TMA samples by immunostaining. Each column represents a single tumor. Samples and immunostaining features were clustered based on Gower distances.

Supplementary Table 1. Summary of *NF1* alterations identified on next-generation sequencing in institutional cohort of 542 glioblastoma. Supplementary Table 2. Genomic correlates of *NF1* alterations within institutional cohort of 542 glioblastoma. Only genes altered at a frequency > 5% were assessed. P-values were calculated with two-sided Fisher exact test. Supplementary Table 3. Genomic correlates of *NF1* alterations within TCGA glioblastoma cohort. Only genes altered at a frequency > 5% were assessed. P-values were calculated with two-sided Fisher exact test. Supplementary Table 4. Genomic correlates of *NF1* alterations within CPTAC glioblastoma cohort. Only genes altered at a frequency > 5% were assessed. P-values were calculated with two-sided Fisher exact test. Supplementary Table 5. Immunohistochemical correlates of *NF1* genomic status in TMA cohort. Comparisons between categorical variables were performed using the Fisher’s Exact test, and two sample independent t-test was used to compare p53 and Ki67 % positivity between conditions. Supplementary Table 6. Immunohistochemical correlates of *NF1* immunostaining in TMA cohort. Comparisons between categorical variables were performed using the Fisher’s Exact test. Supplementary Table 7. Baseline clinicodemographic features of TMA cohort and NF1 immunostaining subgroups. Supplementary Table 8. Univariable and multivariable Cox proportional hazards analysis of overall survival in TMA cohort. Supplementary Table 9. Genomic correlates of NFC immunostaining in subset of *NF1*-wildtype glioblastomas. Only genes altered at a frequency > 5% were assessed. P-values were calculated with two-sided Fisher exact test. Supplementary Table 10. IHC correlates of NFC immunostaining in subset of *NF1*-wildtype glioblastomas. Supplementary Table 11. Univariable and multivariable Cox proportional hazards analysis of overall survival in *NF1*-wildtype subset of TMA cohort.

## Data Availability

Next generation sequencing and tissue microarray data is available upon request from the corresponding author as consent for public data sharing was not obtained from JHH patients.
