# Supplementary figures and images for: NF1 expression profiling in IDH-wildtype glioblastoma: genomic associations and survival outcomes
Source: Acta Neuropathol Commun. 2024 Oct 29;12:172. doi: 10.1186/s40478-024-01875-z (PMC11520828; doi:10.1186/s40478-024-01875-z)

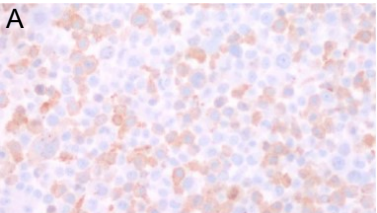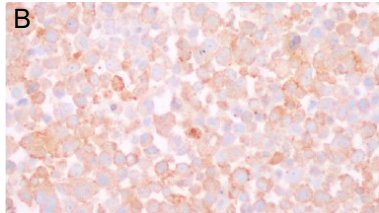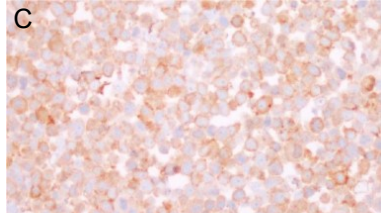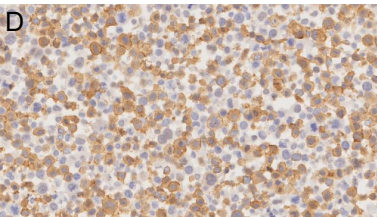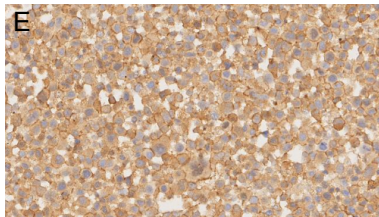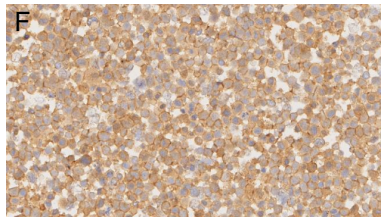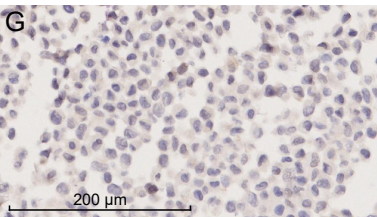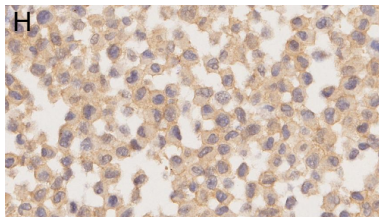

Supplement: Supplementary file 1 — Supplementary Figure 1. IHC validation of NF1 antibodies. (A) Absent NF1 immunostaining with NFC antibody in JHH-520 (NF1 -/-) neurosphere cells mixed with NF1-intact B76 filler cell line. (B,C) Retained NF1 immunostaining with NFC in GBM1 and JHH-0879 neurosphere cell pellets, both with intact NF1. (D) Absent NF1 immunostaining with iNF-07E antibody in JHH-520 neurosphere cells mixed with NF1-intact B76 filler cell line. (E,F) Retained NF1 immunostaining with iNF-07E antibody in GBM1 and JHH-0879 neurosphere cell pellets admixed with NF1-intact B76 filler cell line. (G) Absent and (H) retained NF1 immunostaining with iNF-07E antibody in isogenic NF1 -/- and NF1 +/+ immortalized human Schwann cells respectively. [file 40478_2024_1875_MOESM1_ESM.pdf]

A

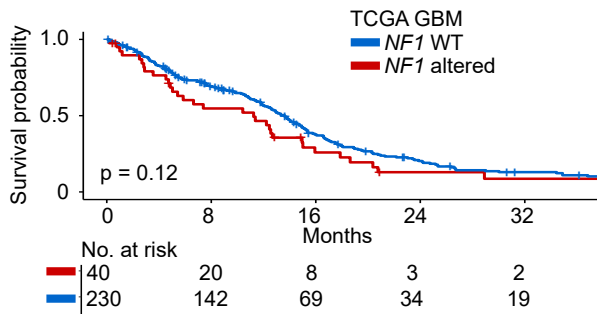

B

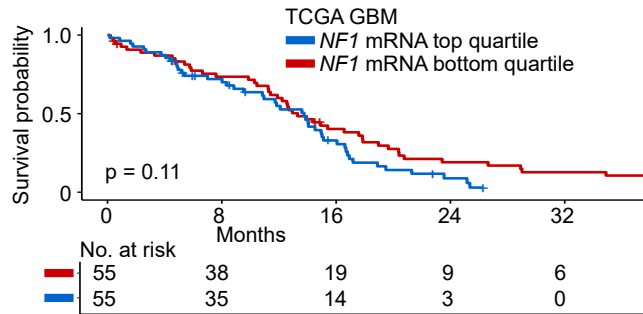

C

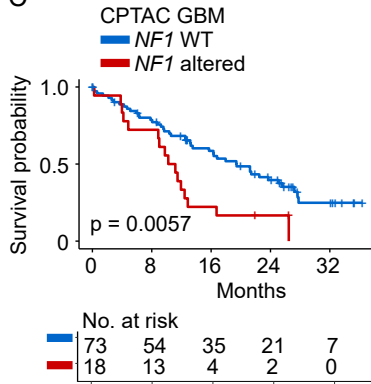

D

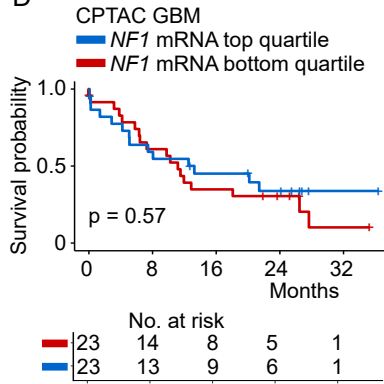

E

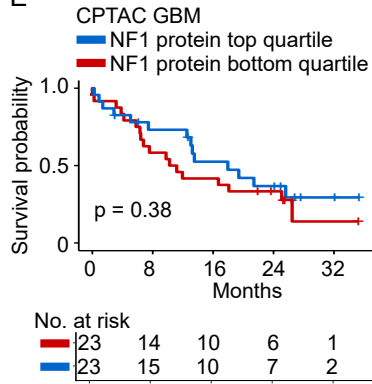

Supplement: Supplementary file 2 — Supplementary Figure 2. Overall survival and NF1 alterations, mRNA, and protein expression in TCGA and CPTAC GBM cohorts. Kaplan-Meier curves displaying overall survival in TCGA cohort stratified by (A) presence of NF1 alteration and (B) NF1 mRNA expression (U133 microarray)– bottom quartile versus top quartile. Kaplan-Meier curves displaying overall survival in CPTAC cohort stratified by (C) presence of NF1 alteration, (D) NF1 mRNA expression – bottom quartile versus top quartile, and (E) NF1 protein expression – bottom quartile versus top quartile. [file 40478_2024_1875_MOESM2_ESM.pdf]

**NF1 alteration**

○ Truncating

○ Missense

**NF1 Immunostaining**

● NFC lost

● Both lost

● Both retained

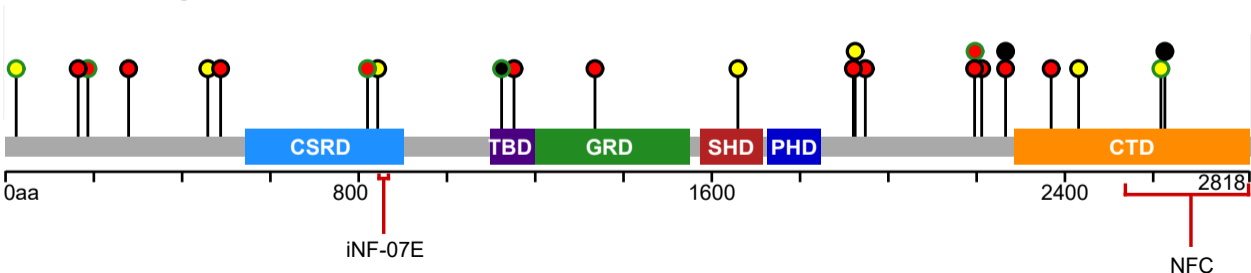

Supplement: Supplementary file 3 — Supplementary Figure 3. Lollipop plot of NF1 alterations identified in TMA cohort with labeled NF1 immunohistochemistry results. iNF-07E antibody epitope and NFC antibody immunogen are labeled in brackets (red). [file 40478_2024_1875_MOESM3_ESM.pdf]

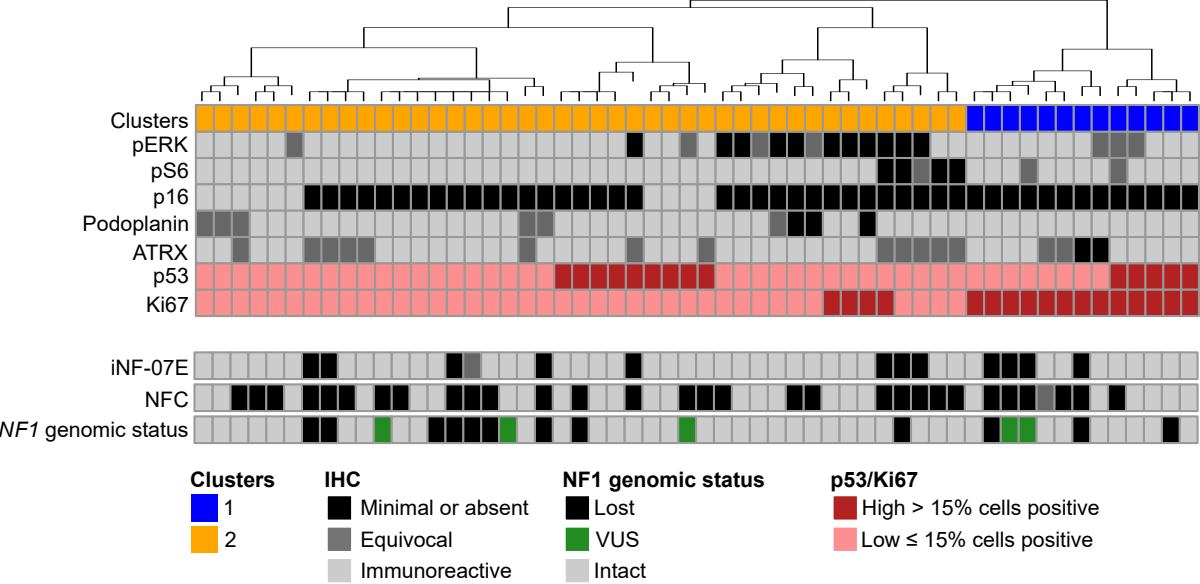

Supplement: Supplementary file 4 — Supplementary Figure 4. Unsupervised hierarchical clustering of TMA samples by immunostaining. Each column represents a single tumor. Samples and immunostaining features were clustered based on Gower distances. [file 40478_2024_1875_MOESM4_ESM.pdf]
